# Fetal thyrotoxicosis after total thyroidectomy due to Graves’ disease

**DOI:** 10.1007/s00404-023-06994-x

**Published:** 2023-04-25

**Authors:** R. Reineke, U. Gembruch, A. Geipel

**Affiliations:** Department of Obstetrics and Prenatal Medicine, University of Bonn, Bonn, Germany

**Keywords:** Graves’ disease, Fetal goiter, Hydrops

## Description

A 33-year-old gravida 3 para 2 was referred at 22 + 2 weeks of gestation for fetal tachycardia. Five years ago, she had a total thyroidectomy due to Graves’ disease. According to the patient’s history, she had given birth to a healthy girl and suffered from unexplained intrauterine demise at 28 + 1 weeks during her second pregnancy.

**Fig. 1:**
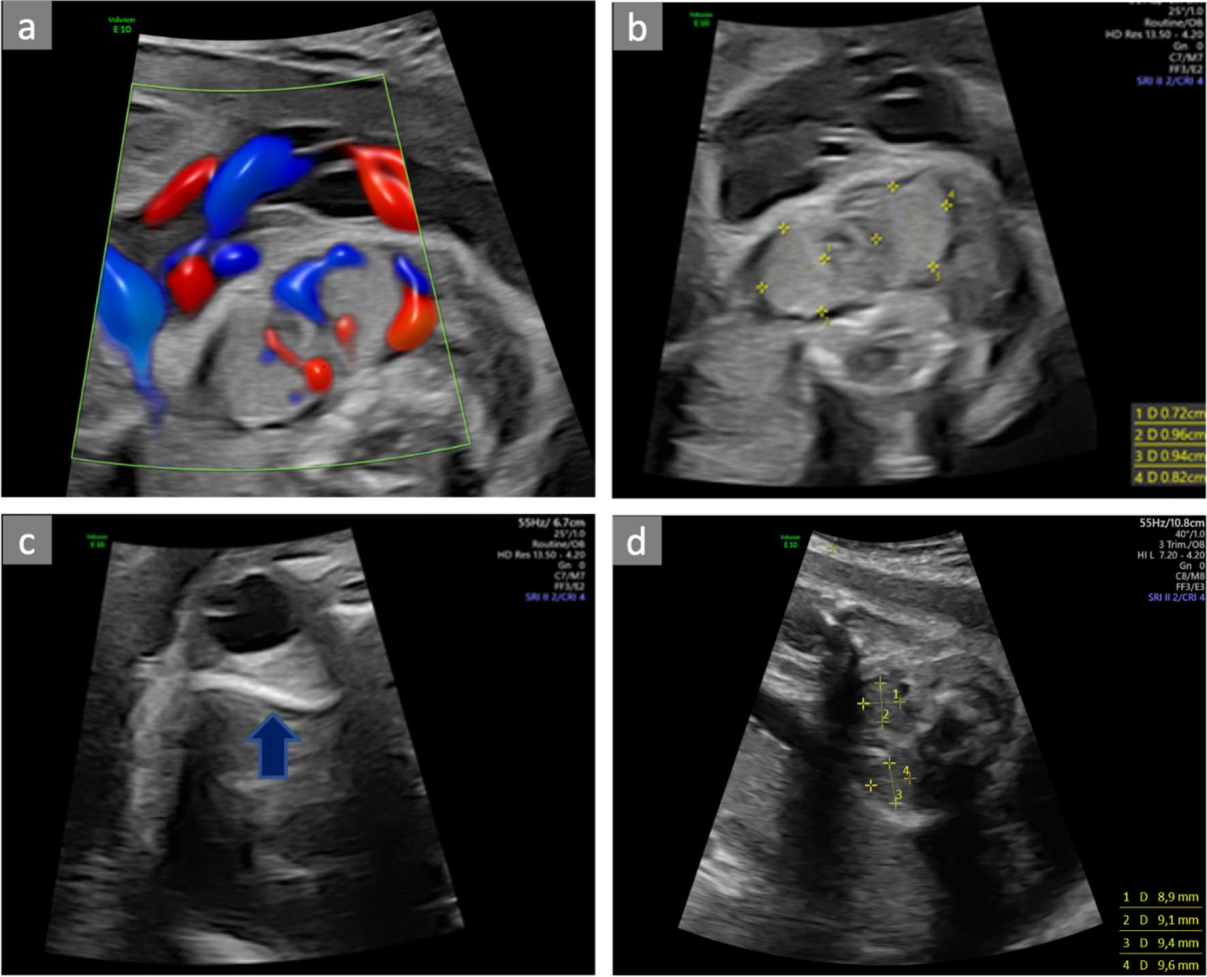
**a, b** Transverse plane of the thyroid gland of the fetus. **a** Color Doppler imaging demonstrates the increased blood flow to the enlarged glands despite high PRF. **b** Biometry shows the enlagerment of thyroid glands. **c** Image of the exophthalmus showing the enlarged area behind the left bulbus (blue arrowhead). **d** Image of the thyroid gland in transverse plane at 26 + 4 weeks. Usually, further growth of the thyroid gland would be expected [4]. However, as a result of successful treatment with PTU, the gland´s size remains constant (95th percentile). By 29 + 4 weeks, the thyroid diameter was within normal range again

Fetal sonography showed goiter, hydrops (bilateral pleural effusions, mild ascites, polyhydramnios), sustained tachycardia of 180/min and cardiomegaly (Fig. 1). Cordocentesis revealed severe fetal hyperthyroidism (TSH 0.01 μU/ml, fT3 5.43 pg/ml, fT4 7.02 ng/dl) and elevated TSH receptor antibodies (TRAb 29.3 IU/l). The pregnant woman herself showed euthyroid values with a daily intake of 175 µg levothyroxine and a high level of TRAb (TRAb 219 IU/l). We initiated therapy with 400 mg propylthiouracil (PTU) daily perorally.

The follow-up sonographies showed rapid normalization of heart rate and remission of hydrops, and, with a time delay, the size of the goiter and the exophthalmos, therefore PTU dosage was lowered to 200 mg daily. A second cordocentesis performed at 29 + 3 weeks showed normal levels of fT3 and fT4 (TSH 0.01 μU/ml, fT3 2.29 pg/ml, fT4 1.01 ng/dl). The patient gave birth at 36 + 6 weeks (3060 g, Apgar score: 8/9/10, umbilical arterial pH: 7.36).

Graves´ disease is rare and affects about 0.2% of pregnant women [5]. TRAb are detected in 95% of Graves´ disease patients [3] and in 30% they are still found after thyroidectomy [1]. As IgG antibodies, TRAb are transplacentally transported and can lead to fetal hyperthyroidism, resulting in sinus tachycardia, goiter, exophthalmos, high cardiac output failure, hydrops and even death [5]. The first sonographic findings are fetal goiter and/or tachycardia. However, a goiter can rarely also be caused by fetal hypothyroidism. Therefore, performing cordocentesis is the diagnostic key [2]. Antithyroid drugs are the therapy of choice, with PTU being the preferred medication as it causes less fetotoxicity [1]. The dose should be chosen to avoid drug-induced fetal hypothyroidism. Sometimes, propranolol needs to be administered additionally to lower the fetal heart rate more quickly [5]. Without treatment, fetal hyperthyroidism is associated with a high fetal morbidity and mortality [3].

## Data Availability

Data available on request from the authors.
